# Dr. John C. Lewis

**Published:** 1897-03

**Authors:** 


Dr. John C. Lewis, of Panama, Chautauqua county, N. Y., died of pneumonia, at his residence in that village, February 6, 1897, aged 48 years.

Dr. Lewis was born at Ellery, N. Y., August 6, 1848, was a graduate of the Westfield Academy, and took his doctorate degree at the medical department of the University of Buffalo in the class of 1874. He settled at Panama soon after graduation, and has been the leading physician in that vicinity for many years.

He was an active member of the Medical Society of the County of Chautauqua, his name having appeared on nearly every program for years. He was a member of the Methodist Episcopal Church; he was also a member of several beneficiary orders. He took a deep interest in his profession and was also a student of botany and astronomy. He married Sarah E. Brownell, March 3, 1870, who, with three children, survives him. Dr. Lewis, at the time of his death, was editor of the *Panama Herald*, and one of the most enterprising men in the community. The medical profession of western New York, in his death, has lost one of its ablest members and the community one of its best citizens. C. A. E.

